# Bias and generalizability of brain age prediction models: A multi-cohort evaluation with anatomical and interpretability insights

**DOI:** 10.1162/IMAG.a.1164

**Published:** 2026-03-12

**Authors:** Lautaro J. Aguzin Parrilli, Martin A. Belzunce

**Affiliations:** Centro Universitario de Imágenes Médicas (CEUNIM), Escuela de Ciencia y Tecnología, Universidad Nacional de Gral. San Martín, Buenos Aires, Argentina; Instituto de Ciencias Físicas (ICIFI UNSAM-CONICET), Escuela de Ciencia y Tecnología, Universidad Nacional de Gral. San Martín (UNSAM), Buenos Aires, Argentina; Consejo Nacional de Investigaciones Científicas y Tecnológicas (CONICET), Buenos Aires, Argentina

**Keywords:** brain age prediction, brain age gap (BAG), deep learning, neuroimaging, aging, interpretability

## Abstract

Brain age prediction from T1-weighted MRI and its associated brain age gap (BAG) has emerged as a promising neuroimaging biomarker for assessing deviations from normative aging. However, the robustness, bias, and interpretability of existing models across external datasets remain poorly understood, limiting clinical translation. In this study, we evaluated four publicly available brain age models (ENIGMA, DeepBrainNet, Pyment, and BrainAgeNeXt) across four independent MRI datasets (ADNI, UNSAM Long COVID, and two OpenNeuro cohorts), comprising 1,634 subjects with diverse demographic and clinical profiles. Models were tested using their original preprocessing pipelines, and performance was assessed using mean absolute error (MAE), mean error (ME), and BAG variability metrics, with additional analyses of biases related to age, dataset, ethnicity, and education. Interpretability was evaluated using Layer-wise Relevance Propagation, and anatomical correlates were explored using BrainChart-derived centile scores. Group-level comparisons were performed between cognitively normal (CN) individuals and patients with Mild Cognitive Impairment (MCI), Alzheimer’s disease (AD), or Long COVID (LC). Models based on 3D convolutional neural networks (Pyment and BrainAgeNeXt) outperformed the DeepBrainNet 2D CNN and the ENIGMA ridge regression model in both accuracy (MAE: 3.9–3.7 vs. 6.2–12.4 years respectively) and stability (ASTD: 3.2–2.9 vs. 4.6–8.3 years). Dataset-specific BAG differences were largely explained by age distributions, whereas ethnicity showed a statistically significant but small effect on BAG in some models. Relevance maps highlighted the lateral ventricles as the most consistently relevant anatomical region, with additional cerebellar contributions emerging in older adults for BrainAgeNeXt. Group-level analyses confirmed elevated BAG in MCI and AD patients compared to CN, while no significant differences were observed in Long COVID participants. These findings suggest that, while BAG is a promising biomarker for group-level analyses, current models are required to address age and demographic biases to enable individual-level clinical application.

## 1 Introduction

In recent years, artificial intelligence techniques, particularly Machine Learning (ML) and Deep Learning (DL), have been increasingly applied to the study of brain aging (Wu et al., 2024). One prominent application is the estimation of an individual’s brain age from neuroimaging data, most commonly using T1-weighted structural MRI. In this approach, models are trained to predict chronological age from brain imaging-derived features. The difference between predicted and actual age, often referred to as brain age gap (BAG) or predicted age deviation (PAD) (Cole et al., 2017; Cole & Franke, 2017; Franke et al., 2010), can serve as a biomarker of atypical aging or pathology (Cole & Franke, 2017; Singh et al., 2022). A positive BAG value, where the predicted age exceeds chronological age, may reflect neurobiological alterations associated with adverse age-related health outcomes. Conversely, a negative BAG suggests a younger-appearing brain, potentially reflecting better cognitive health and resilience against age-related decline (Elliott et al., 2021; Jawinski et al., 2022).

One of the most significant challenges in developing reliable brain age prediction models is ensuring their robustness and ability to generalize across diverse populations, including variations in age, sex, ethnicity, and socioeconomic status (Ricci Lara et al., 2022). This remains difficult because existing public neuroimaging datasets often lack sufficient demographic and regional diversity (Wu et al., 2024). Several recent works (Glocker et al., 2019; Wachinger et al., 2021) have shown that machine-learning models are heavily biased by the acquisition site, and despite showing promising results on internal validation, many models tend to show a marked drop in performance when tested on external datasets, often systematically underestimating or overestimating brain age.This issue can be attributed to various factors such as differences in scanner manufacturers, specifications, settings and hardware acquisition protocols (Glocker et al., 2019), and demographic distributions (Ricci Lara et al., 2022). As a result, ML models, particularly Deep Neural Networks, tend to overfit site-specific characteristics, even when trained on multi-site datasets (Dufumier et al., 2022).

Therefore, if brain age estimation is to be used as a clinically relevant biomarker, it is essential to develop models that are not only accurate but also robust to variations across sites and populations and provide explainability tools to promote trust, accountability, transparency, and interpretability (Sadeghi et al., 2024).

In recent years, several research groups have released publicly available, pre-trained brain age models that can be readily applied to new datasets following minimal preprocessing steps. While both ML and DL based approaches have been used successfully, recent studies suggest that DL models, particularly those employing Convolutional Neural Networks (CNNs), consistently outperform traditional ML models, especially when tested with external datasets (Dörfel et al., 2023; Wu et al., 2024). Among DL approaches, models that integrate CNNs with transformers-inspired mechanisms (Vaswani et al., 2017) have demonstrated the highest overall model performance (Wu et al., 2024).

In this work, we aim to evaluate the generalizability and bias of publicly available brain age prediction models when applied to external datasets with differing demographics and technical characteristics. While previous studies have explored aspects of this problem (Dörfel et al., 2023; Jirsaraie et al., 2023), our work provides a more in-depth analysis of the underlying sources of bias, by examining age-related, dataset-specific and ethnicity-based deviations, incorporating interpretability through explainability tools, assessing specific brain tissue volumes relative to normative aging trajectories, and performing a group-level analysis to assess BAG as a biomarker of healthy brain aging. To achieve this, we systematically assess and compare the performance of four pre-trained brain age models based on different architectures across four external datasets. Our goal is to inform the development of more robust and clinically reliable brain age prediction frameworks that maintain accuracy across diverse real-world applications.

## 2 Materials and Methods

### 2.1 Datasets

Four external T1-weighted structural MRI datasets from 3T scanners were used to perform brain age predictions:

- A sample of 1124 scans, one per participant, from the Alzheimer’s Disease Neuroimaging Initiative phase 3 (ADNI 3) (Weiner et al., 2017), including subjects classified into three diagnostic subgroups: Cognitively Normal (CN), Mild Cognitive Impairment (MCI) and Alzheimer Disease (AD).
- A proprietary dataset from a study looking at the impact of Long COVID (LC) on the brain (UNSAM_LC) (Cataldo et al., 2024), which included 169 LC patients and 47 healthy controls. LC participants reported persistent cognitive complaints following SARS-CoV-2 infection and were screened to exclude major neurological or psychiatric disorders prior to inclusion in the study. Preliminary results of this study showed no clear cognitive impairment in the LC group compared to matched healthy controls. However, mild regional atrophy was observed in areas such as the cerebellum, postcentral gyrus, and lingual gyrus, among others (Cataldo et al., 2024).
- The RRIB dataset, publicly available in OpenNeuro from a study that investigated the Blood Oxygenation Level Dependent (BOLD) variability and functional connectivity during cognitive control tasks across the adult lifespan over 158 patients (Rieck et al., 2024).
- The JUK dataset from a study investigating the effects of chronotype, sleep quality, and daytime sleepiness on brain structure, includes 136 young healthy adults (Zareba et al., 2022).

**Fig. 1.**
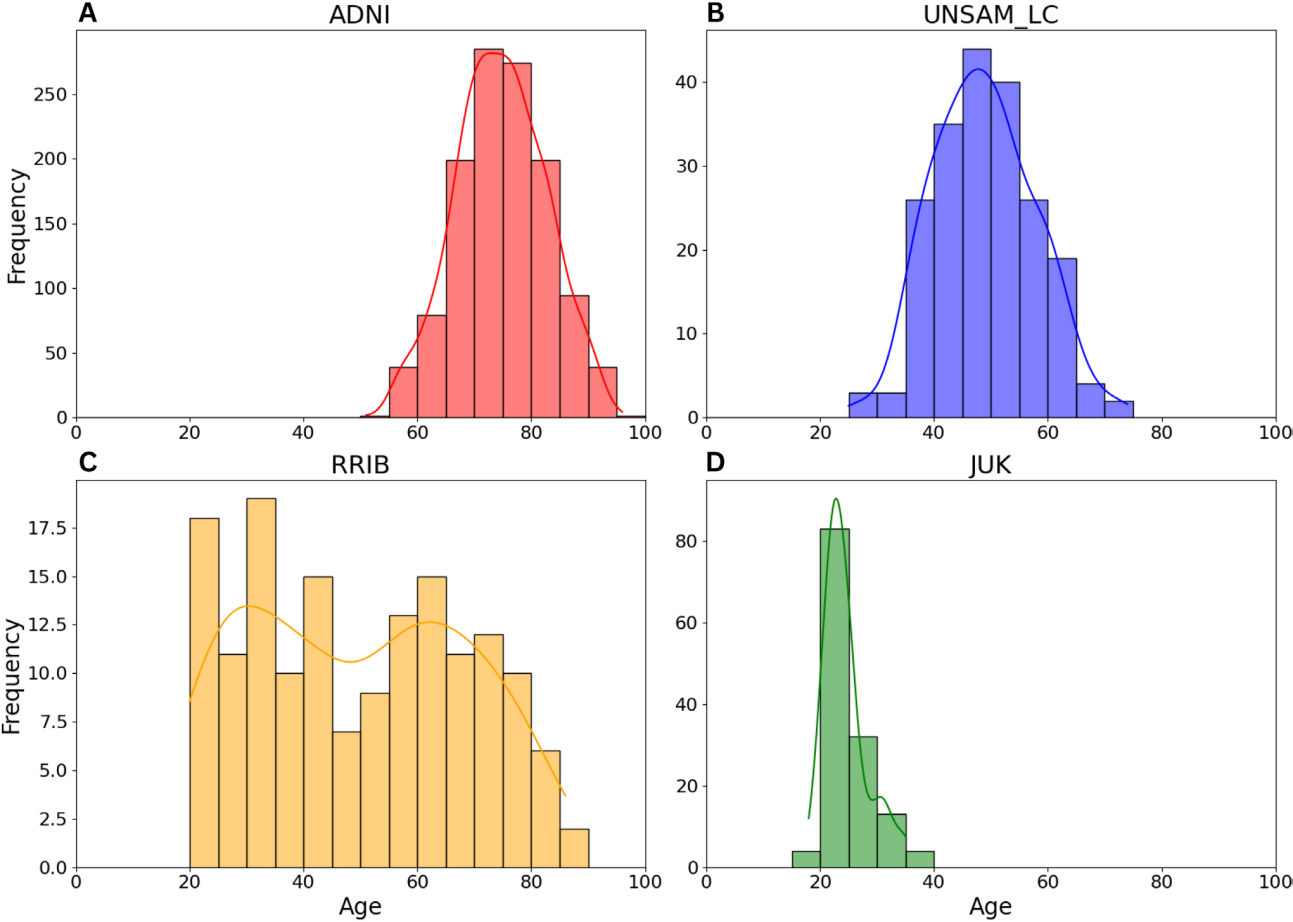
Age distribution histograms for each of the four external MRI datasets used in this study. (A) ADNI (CN, MCI, and AD). (B) UNSAM_LC (Long COVID and matched controls). (C) RRIB (adult lifespan sample). (D) JUK (young healthy adults).

Together, these datasets comprise a total of 1,634 subjects. Demographic information, clinical subgroups, and other relevant variables for each dataset are summarized in Table 1. Figure 1 presents histograms of the age distribution for each dataset, illustrating differences in demographic profiles across cohorts.

**Table 1.** Overview of demographic, clinical, and scanner characteristics of the MRI datasets used for the assessment of brain age prediction models.

Dataset	N subjects	Subgroups	Age [min–max]	Sex (%m)	Scanner	Country	Ethnicities	Years of education
ADNI	535	CN	51–95	40	8 acquisition scanners^*^	United States of America	White (N = 954) –Black (N = 83)- Hisp/Latino (N = 69) –Asian (N = 17)	16 ± 2
	523	MCI	56–96	55				
	65	AD	55–91	62				
UNSAM_LC	47	CN	33–72	42	Siemens Prisma	Argentina	N/A	14 ± 3
	169	LC	25–74	46				
RRIB	158	CN	20–86	37	Siemens Trio	Canada	N/A	N/A
JUK	136	CN	18–35	36	Siemens Skyra	Poland	N/A	N/A

* Scanners from the ADNI 3 dataset include: GE 750, GE 750 W Siemens Prisma/ PrismaFit, Siemens Skyra, Siemens Verio, Siemens Trio/ TIM, Philips Achieva, Philips Ingenia 3T CX and Other/ Undecided.

All T1-weighted scans underwent image quality control using MRIQC (Esteban et al., 2017). From the output, we extracted two key quality metrics: Contrast-to-Noise Ratio (CNR), which reflects tissue contrast quality, and Entropy Focus Criterion (EFC), a measure of image sharpness and motion-related blurring. We used these metrics to screen for outlier scans and to assess potential associations with model prediction error.

### 2.2 Models evaluated

We selected four publicly available brain age prediction models based on the following inclusion criteria: (1) the model had to be publicly available, (2) inclusion of pre-trained weights, and (3) the required preprocessing pipeline had to be specified. The evaluated models were ENIGMA (Han et al., 2021), DeepBrainNet (Bashyam et al., 2020), Pyment (Leonardsen et al., 2022) and BrainAgeNeXt (La Rosa et al., 2025; Liu et al., 2022; Roy et al., 2023). A summary of key model characteristics is presented in Table 2.

**Table 2.** Characteristics of the brain age prediction models included in this study.

Model	Algorithm	Features	Subjects	Preprocessing	Reported accuracy
ENIGMA	Ridge regression	Region based (FreeSurfer)	N = 2188 18–75 years	FreeSurfer’s recon-all pipeline	MAE = 6.7
DeepBrainNet	2D CNN	Voxel based (T1)	N = 11729 3–95 years	N4 bias field correction + skull-stripping + affine MNI 152 registration	MAE = 4.1
Pyment	3D CNN	Voxel based (T1)	N = 53542 3–85 years	Skull-stripping + reorientation + rigid MNI registration + voxel intensity normalization	MAE = 3.9
BrainAgeNeXt	Transformer inspired 3D CNN	Voxel based (T1)	N = 10051 5–95 years	N4 bias field correction + skull stripping + affine MNI 152 registration	MAE = 2.8

#### 2.2.1 ENIGMA

ENIGMA is an ML model developed by the ENIGMA consortium in 2020 based on FreeSurfer-derived features. The model uses ridge regression to estimate brain age and takes as input a set of 77 morphological features derived from the Desikan-Killiany atlas (7 subcortical volumes, 34 cortical thickness regions, 34 cortical surface area regions, lateral ventricles, and intracranial volume). These features are averaged across hemispheres. The model was trained on its own dataset spanning individuals aged 18 to 75 years and provides separate prediction models for males and females. The ENIGMA brain age tool is available as a user-friendly web application: https://photon-ai.com/enigma_brainage

#### 2.2.2 DeepBrainNet

DeepBrainNet is a 2D CNN model published in 2020 that leverages a 2D CNN initialized with ImageNet pre-trained weights. Each T1-weighted MRI scan is processed as a set of 80 axial slices, which are treated as independent inputs during training. At inference time, the model computes the median prediction across slices to generate a subject-level brain age. The model was trained using a large and diverse set of neuroimaging cohorts, with primary contributions from UK Biobank, SHIP, and PNC datasets. The full implementation and pretrained model are publicly available via: https://github.com/vishnubashyam/DeepBrainNet/tree/master

#### 2.2.3 Pyment

Pyment is a lightweight 3D CNN model published in 2022, with a Simple Fully Convolutional Network (SFCN) architecture (Peng et al., 2021). It was trained on data from 21 different datasets, with major contributions from OASIS3, UK Biobank, and FCON1000. The subjects included in the training sample are predominantly within two distinct age ranges: approximately 20 years old and between 50 and 70 years old. It provides 3 model variants, *Soft-max output, Ranking and Regression.* In this work we used the *Regression* variant, which has presented the best performance (Leonardsen et al., 2022). The author provides a pre-trained version of the model, along with tools for explainability and fine-tuning through their GitHub repository: https://github.com/estenhl/pyment-public?tab=readme-ov-file#publications

#### 2.2.4 BrainAgeNeXt

BrainAgeNeXt, published in 2025, is a 3D CNN based on the MedNeXt Architecture and inspired by transformer designs. The model was trained on data from 12 different datasets, with primary contributions from OpenBHB and OASIS-4. The training sample predominantly includes adolescents, young adults, and in a lower proportion older individuals around 70 years of age. BrainAgeNeXt is publicly available via: https://github.com/FrancescoLR/BrainAgeNeXt/tree/main?tab=readme-ov-file

### 2.3 Data pre-processing

To evaluate each model under optimal conditions, the T1-weighted images from the selected datasets were preprocessed according to the specific pipeline recommended by each method (see Table 2). This approach was chosen to maximize model performance and minimize errors due to preprocessing mismatches.

For ENIGMA, we used FreeSurfer’s recon-all to extract cortical and subcortical measures as required, for all the subjects of every dataset. For DeepBrainNet, the original pipeline recommends MASS for skull-stripping, which was not available. We compared BET and SynthStrip as alternatives, as well as ANTsPyNet DeepBrianNet full implementation, and adopted BET based on superior MAE performance (see Supplementary Figure S2 and Supplementary Table S2).

For Pyment and BrainAgeNeXt, preprocessing followed the original recommendations. For the former, skull-stripping was achieved using FreeSurfer, followed by rigid registration to 1 mm MNI space using FSL’s FLIRT, and voxel intensity normalization. For BrainAgeNeXt, the preprocessing pipeline included N4 bias field correction, skull stripping using FreeSurfer’s SynthStrip, and affine registration to the 1 mm MNI152 template using ANTs’ antsRegistrationSyN tool.

### 2.4 Model evaluation strategy

#### 2.4.1 Metrics

The output of each model was the predicted brain age, from which we computed the Brain Age Gap (BAG), a widely used biomarker of atypical aging. It is defined as:

$$BAG_{i}\;=\;Predicted\;Age_{i}\;-\;Chronological\;Age_{i}\;$$ Eq. 1

To evaluate model performance, we used four complementary metrics that measure accuracy, biases and variability of the age predictions:

- Mean Absolute Error (MAE). Quantifies overall prediction accuracy:
  $$MAE\;=\;\frac{1}{N}\sum_{i}^{N}\left|BAG_{i}\right|\;\;$$ Eq. 2
- Mean Error (ME). Measures systematic biases in the predictions:
  $$ME\;=\;\frac{1}{N}\sum_{i}^{N}BAG_{i}\;\;\;$$ Eq. 3
- Standard Deviation of BAG (STD). Measures the dispersion of individual BAG values around the ME:
  $$STD\;=\;\sqrt{\frac{\sum_{i=1}^{N}{\left(BAG_{i}-\;ME\right)}^{2}}{N}}\;$$ Eq. 4
- Absolute BAG standard deviation (ASTD). Evaluates the variability of individual absolute BAG around the MAE:
  $$ASTD\;=\;\sqrt{\frac{\sum_{i=1}^{N}{\left({\left|BAG_{i}\right|}_{i}-\;MAE\right)}^{2}}{N}}\;$$ Eq. 5

In all formulas, *i* indices the individual subjects (from i=1 to N), where N is the total number of subjects evaluated. *BAG_i_* represents the brain age gap for subject i, which is expected to average around zero for healthy individuals in an accurate model.

#### 2.4.2 Accuracy evaluation

MAE was first computed for all CN subjects within each dataset to assess model accuracy under realistic, dataset-specific conditions. For all models, the full set of CN subjects was used. To visually inspect model performance, scatter plots of predicted brain age versus chronological age were generated for each model, where deviations from the identity line indicate systematic over- or underestimation.

Additionally, to evaluate model robustness across datasets while accounting for sample size differences, we computed balanced global MAE and ASTD metrics for each model using 20 random subsamples of 47 cognitively normal participants per dataset, drawn without replacement. This number corresponds to the maximum number of CN available in the LC cohort.

#### 2.4.3 Bias and robustness

##### 2.4.3.1 Age and dataset related bias

ME was also first computed for all CN subjects within each dataset to assess systematic prediction bias and variability, and then a balanced global ME and STD were computed the same way as for the balanced global MAE and ASTD.

To evaluate age-related bias and prediction agreement, we generated Bland–Altman plots for each model and dataset. These plots display the BAG as a function of the average between predicted and chronological age. A univariate linear regression line was fitted to each plot to assess trends such as regression-to-the-mean effects, age-dependent errors, or outliers.

Lastly, to disentangle whether potential biases in BAG values reflect age-related or dataset-specific effects, we fitted a multivariate linear regression model using demeaned chronological age as a continuous predictor and dataset as categorical predictors, where we also included age-dataset interaction terms. The RRIB dataset was selected as the reference group given its broad age range distribution. The fitted model was:

$$BAG_{i}=\beta_{0}+\beta_{1}\cdot Age_{i}+\sum_{j=2}^{4}\beta_{j}\cdot D_{ji}+\sum_{j=2}^{4}\gamma_{j}\left(Age_{i}\cdot D_{ji}\right)\;$$ Eq. 6

Where *BAG_i_* is the brain age gap for subject *i*, *Age_i_* is the demeaned chronological age, and $D_{2i}$ , $D_{3i}$ , and $D_{4i}$ are binary variables indicating whether subject *i* belongs to the ADNI, UNSAM_LC, or JUK datasets, respectively. β are the linear regression coefficients and γ the coefficients for the interaction terms.

This model allows us to estimate and test for dataset-specific shifts in BAG, while adjusting for age. A significant age term ($\beta_{1}$) would indicate age-related bias in the model’s predictions, whereas significant dataset terms ($\beta_{2}$, $\;\beta_{3}$ and $\beta_{4}$) would reflect systematic differences in BAG across datasets, independent of age. Finally, significant interaction terms ($\gamma_{2}$, $\gamma_{3}$ and $\gamma_{4}$) would indicate that the relationship between BAG and age differs across datasets, revealing dataset-specific age-related biases.

We fitted the proposed linear regression to the predictions of each brain age prediction model. For each predictor variable of the regression, we report coefficient estimates, statistical significance (*p*-values), interaction’s statistical significance (interaction *p-*values) effect sizes (Cohen’s f²), and coefficients of determination R^2^. For the Cohen’s f² effect sizes, we used the following criteria: 0.02: small, 0.15: medium, 0.35: large effects.

##### 2.4.3.2 Ethnicity and education related bias

To evaluate potential ethnicity-related bias, we examined whether ethnicity had a significant influence on BAG values among cognitively normal participants from the ADNI cohort, the only dataset with available ethnicity information. Ethnicity categories included were White, Black, Hispanic and Asian, as they were the only with more than 10 subjects. BAG distributions for each ethnicity were visualized using boxplots. An analysis of covariance (ANCOVA) was then performed with ethnicity as a categorical factor while controlling for age. We report the F-statistic, *p*-value, and Cohen’s f² for both predictors. For models showing a significant effect of ethnicity, we conducted a post-hoc Tukey test to identify specific pairwise group differences.

To assess the effect of years of education, we fitted a multivariate linear regression using years of education, age, and clinical group as predictors of BAG. This analysis was performed separately for ADNI and UNSAM_LC datasets, the only datasets for which education data were available.

#### 2.4.4 Interpretability

To investigate which brain regions contribute most to the final brain age prediction, we employed a Layer-wise Relevance Propagation (LRP) algorithm as an explainability method. LRP highlights relevant voxel-level areas in the input images that either support or oppose the model’s output decision (Bach et al., 2015; Montavon et al., 2018). This analysis was conducted on models based on 3D CNNs architectures, specifically BrainAgeNeXt and Pyment, for CN subjects.

For Pyment, we used the original LRP implementation provided by the authors, which is custom-tailored to their architecture. For BrainAgeNeXt, we adapted the original prediction script to generate relevance maps using the Zennit library (Anders et al., 2021) within the PyTorch framework. Subject-level relevance maps were averaged within each dataset and resampled to MNI space. Using the Hammer’s atlas (Hammers et al., 2003), we quantified regional relevance by averaging voxel-wise relevance scores within each anatomical region of interest (ROI). For visualization, we show relevance maps across axial slices (z = 20 to 120, step = 20) for the ADNI, UNSAM_LC, and JUK datasets, accounting for differences in age distributions.

Previous studies have consistently reported that periventricular and subcortical regions, particularly the lateral ventricles, are highly relevant in brain age prediction models (Hepp et al., 2021; Hofmann et al., 2022). Motivated by these findings and the average relevance scores obtained for each ROI, we complemented our relevance map analysis by exploring whether deviations from normative ventricular and brain tissue volumes could relate to model biases. Specifically, we examined ventricular, cortical gray matter, subcortical gray matter, and white matter volumes using BrainChart, a normative modeling framework that provides age- and sex-adjusted centile scores (Bethlehem et al., 2022).

For each subject, we derived centile scores for the major tissue compartments, measured with freesurfer and computed median centile values per subgroup and dataset. Values above or below the 50th centile were interpreted as relative enlargement or reduction compared to normative aging trajectories. This exploratory analysis aimed to contextualize model predictions in terms of structural brain variation across datasets.

#### 2.4.5 Group-level differences

To assess the utility of BAG as a clinical biomarker, we evaluated whether clinical groups (MCI, AD from ADNI and Long COVID from UNSAM_LC) exhibit significantly higher BAG values compared to CN individuals from the same dataset. For BAG to be clinically meaningful, we established the following expectations for each group:

- Cognitively Normal (CN) individuals: BAG values are expected to be close to zero, reflecting unbiased predictions.
- MCI and AD: BAG values are expected to be positive and higher compared to CN individuals from the same dataset, suggesting accelerated brain aging associated with neurodegenerative processes.
- Long COVID: BAG values are hypothesized to be near zero, consistent with their generally normal cognitive profiles (Cataldo et al., 2024). However, based on previous findings of mild cortical atrophy and other structural brain changes in some Long COVID cohorts (Cataldo et al., 2024; Douaud et al., 2022; Nabizadeh et al., 2024), slightly elevated BAG values may also be expected.

To formally test group-level differences in BAG between clinical groups, we performed an ANCOVA, with chronological age included as a covariate to account for age-related bias. When the group effect reached statistical significance, post-hoc pairwise comparisons were performed using Tukey’s Honest Significant Difference test.

## 3 Results

### 3.1 Image quality assessment

All scans passed the image quality control, and no scans exhibited outlier values on CNR or EFC based on the standard MRIQC thresholds. Mean CNR was 2.94 ±  0.53, 3.61 ± 0.32, 2.61 ± 0.53, and 3.13 ± 0.25, for ADNI, UNSAM_LC, RRIB and JUK respectively. Mean EFC was 0.57 ± 0.07, 0.57 ± 0.03, 0.55 ± 0.02, and 0.56 ± 0.04, for ADNI, UNSAM_LC, RRIB, and JUK respectively. Histograms with CNR and EFC distributions are available in Supplementary Figure S1 of the Supplementary Material.

### 3.2 Accuracy evaluation

Table 3 reports the MAE across models and datasets for cognitively normal subjects. BrainAgeNeXt demonstrated the highest overall accuracy in terms of MAE, and specifically for the ADNI, RRIB and JUK datasets, while Pyment showed the best performance on the UNSAM_LC dataset. In contrast, ENIGMA exhibited the highest MAE across all datasets.

**Table 3.** Mean absolute error (MAE) for each mode across external validation datasets (CN subjects only), and balanced global MAE (mean ± SD) computed from 20 random subsamples of 47 CN participants per dataset.

	Reported MAE [years]	ADNI [years]	UNSAM_LC [years]	RRIB [years]	JUK [years]	Balanced global MAE [years]^*^	Balanced global ASTD [years]^*^
ENIGMA	6.7	13.8	7.4	16.4	12.7	12.4 ± 0.4	8.3 ± 0.5
DeepBrainNet	4.1	5.9	4.6	6	8.4	6.2 ± 0.2	4.6 ± 0.3
Pyment	3.9	5.3	3.7	3.8	2.9	3.9 ± 0.2	3.2 ± 0.2
BrainAgeNeXt	2.8	4	4.6	3.5	2.5	3.7 ± 0.2	2.9 ± 0.2

* Balanced global MAE and ASTD were computed by averaging 20 random subsamples of 47 cognitively normal participants per dataset, drawn without replacement.

In terms of image quality, neither CNR nor EFC showed significant correlations with the model’s MAE across any dataset or model (see Supplementary Table S1 of the Supplementary Material), suggesting that variations in image quality did not substantially influence performance metrics in this study.

**Fig. 2.**
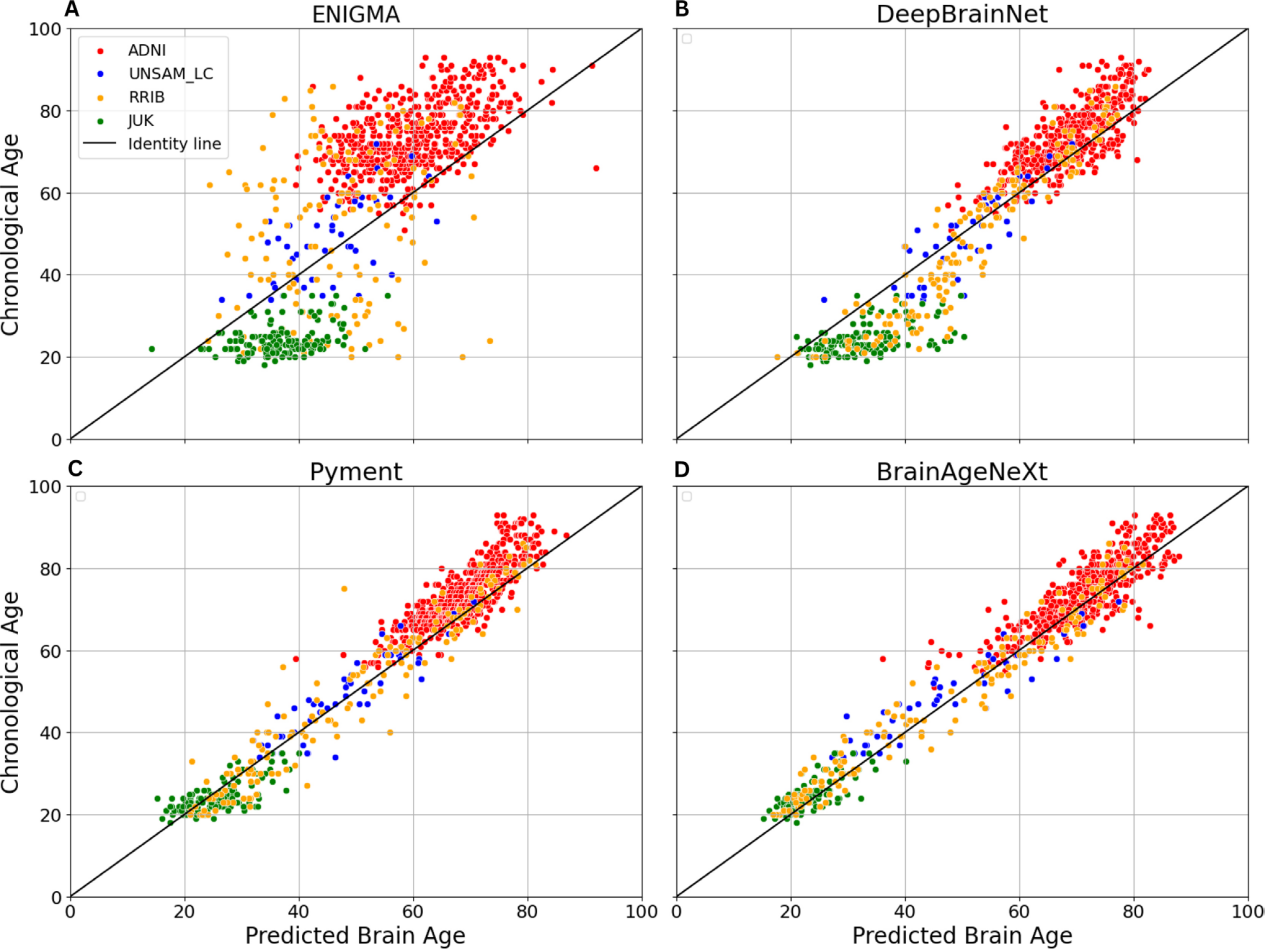
Scatter plots of Predicted Brain Age vs Chronological Age for the four models under analysis when tested with CN Subjects from all datasets (identified with different colors). (A) ENIGMA. (B) DeepBrainNet. (C) Pyment. (D) BrainAgeNeXt.

To enable visual inspection of model predictions, Figure 2 shows scatter plots of predicted versus chronological age for each model and dataset. The black identity line represents perfect prediction. Points on the left of the line indicate underestimation of age, while points on the right indicate overestimation. The ENIGMA model, in addition to the highest error in brain age estimations, shows a considerable regression-to-the-mean effect, as predictions are biased toward the central tendency of the training set age distribution, between 40 and 60 years old.

### 3.3 Bias and robustness

#### 3.3.1 Age- and dataset-related bias

Table 4 presents balanced global ME and STD for each model, and ME values stratified by dataset. Pyment exhibited the lowest balanced global ME, indicating minimal systematic bias and the most balanced performance in terms of over- and under-estimation. Moreover, both BrainAgeNeXt and Pyment achieved the lowest balanced global STD, suggesting more consistent predictions across individuals.

**Table 4.** Mean error (ME) for each model across external validation datasets (CN subjects only), and balanced global ME (mean ± SD) computed from 20 random subsamples of 47 CN participants per dataset.

	ADNI [years]	UNSAM_LC [years]	RRIB [years]	JUK [years]	Balanced global ME^*^ [years]	Balanced global STD^*^ [years]
ENIGMA	-13.6	-3.3	-4.2	12.6	-2.2 ± 0.9	14.8 ± 0.5
DeepBrainNet	-4.8	1.7	2.8	8.2	1.8 ± 0.3	7.4 ± 0.3
Pyment	-4.9	-1.0	-0.2	1.4	-1.2 ± 0.2	4.9 ± 0.2
BrainAgeNeXt	-2.6	-2.0	-0.9	-1.5	-1.7 ± 0.2	4.3 ± 0.2

* Balanced global ME and STD were computed by averaging 20 random subsamples of 47 cognitively normal participants per dataset, drawn without replacement.

Models based on CNNs demonstrated lower ME across most datasets. However, pronounced dataset-specific biases were observed. In particular, predictions for the ADNI dataset (composed mainly of older subjects) tended to underestimate brain age, whereas predictions for the JUK dataset (younger individuals) tended to overestimate it.

For DeepBrainNet, here we show the results for the original implementation and preprocessing with FSL’s BET for brain extraction. In Section S2 of the Supplementary Material, we additionally show MAE (Supplementary Table S2) and ME (Supplementary Table S3) results for FreeSurfer’s SynthStrip and for the full implementation available in the ANTsPyNet package.

**Fig. 3.**
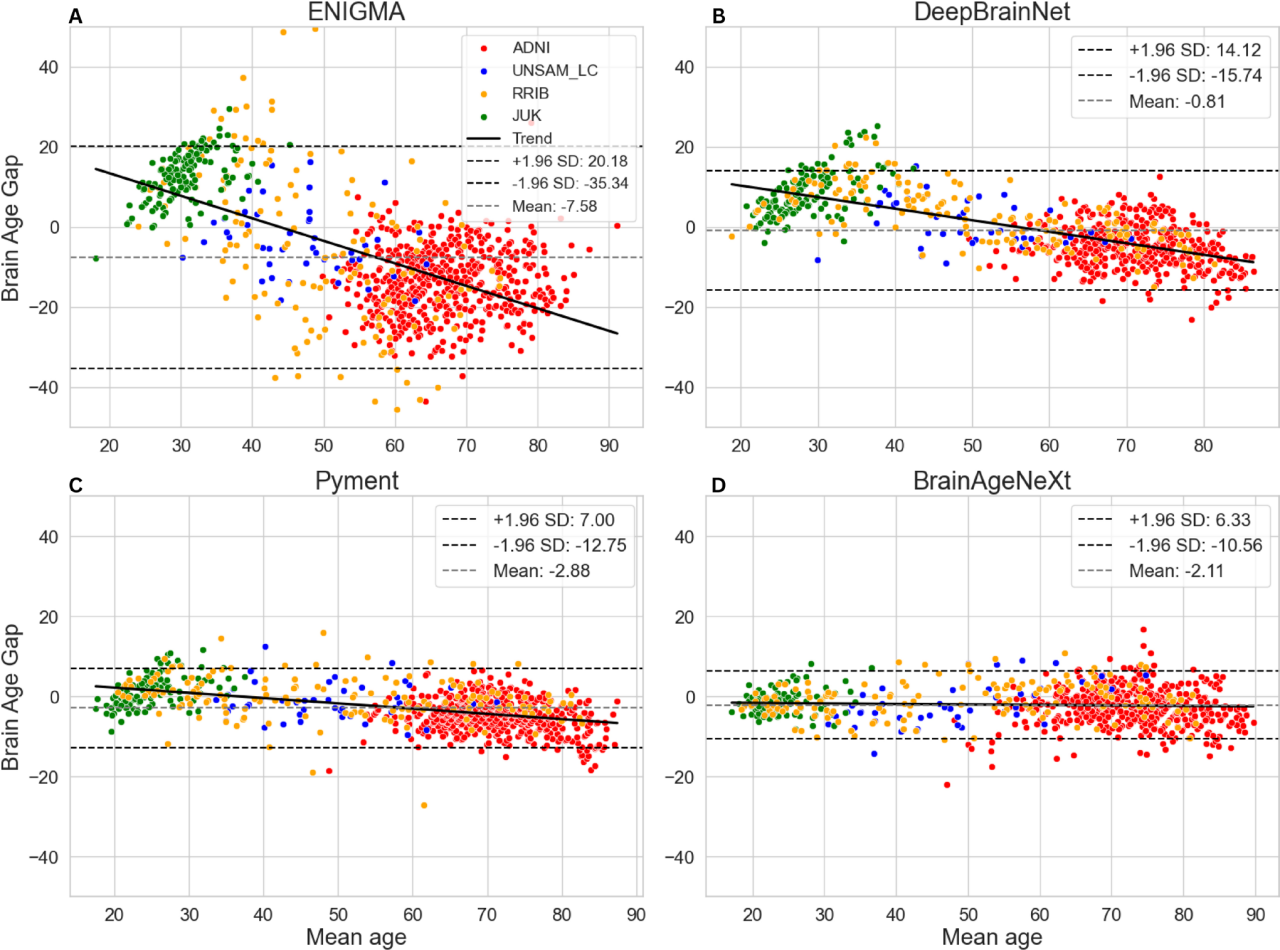
Bland-Altman plots for the four models (A: ENIGMA. B: DeepBrainNet. C: Pyment. D: BrainAgeNeXt), using predictions of CN subjects from all datasets. The y axis represents the prediction error (which is equal to BAG for healthy individuals), and the x axis is the mean of the chronological and predicted ages. The linear regression represented with the black line was computed across all subjects.

Figure 3 shows Bland-Altman plots for each model and dataset, where it becomes evident that, across all datasets and models, there is a systematic trend whereby models tend to overestimate the brain age in younger subjects and underestimate it in older ones, as the regression slope is negative in all cases. This trend is also apparent in Table 5, where ME values decrease across successive age decades for all models.

**Table 5.** Mean error reported across decade-stratified groups for the full CN sample, for all brain age prediction models.

Model	20–29 [years]	30–39 [years]	40–49 [years]	50–59 [years]	60–69 [years]	70–79 [years]	80–89 [years]
ENIGMA	14.3	8.0	-1.1	-6.3	-11.2	-15.0	-18.3
DeepBrainNet	8.6	7.6	4.0	-1.3	-1.9	-4.6	-8.4
Pyment	1.8	0.7	-0.5	-1.9	-2.8	-4.3	-6.9
BrainAgeNeXt	-1.3	-2.6	-2.1	-1.7	0.8	-1.9	-4.4

When comparing models, DeepBrainNet and ENIGMA showed the most pronounced overestimation in young subjects and underestimation in older ones. This effect was milder for Pyment, while BrainAgeNeXt exhibited a relatively flat bias across the lifespan, albeit with consistent underestimation of brain age.

To quantify the contributions of age and dataset to BAG, we fitted a multivariate linear regression using the demeaned chronological age and dataset as predictors (Eq. 6). Table 6 shows the regression coefficients, associated *p*-values, interactions *p*-values, effect sizes (Cohen’s *f²*), and R^2^ for each model.

**Table 6.** Coefficients, *p*-values, interaction-term *p*-values (*p*_$\gamma_{\text{i}}$), and R² from multivariate regressions (Eq. 6) for each model, assessing the influence of demeaned age and dataset (RRIB as reference) on BAG in CN subjects.

	Intercept		Age		ADNI			UNSAM_LC			JUK			Age effect size	Dataset effect size	
Model	$\beta_{0}$	*p*	$\beta_{1}$	*p*	$\beta_{2}$	*p*	*p_ $\gamma_{2}$*	$\beta_{3}$	*p*	*p_$\gamma_{3}$*	$\beta_{4}$	*p*	*p_$\gamma_{4}$*	Cohen’s f²	Cohen’s f²	R²
ENIGMA	-13.48	<0.01	-0.85	<0.01	5.67	<0.01	<0.01	3.81	0.08	<0.01	13.66	0.06	<0.01	1.27	0.18	0.74
DeepBrainNet	0.00	0.99	-0.28	<0.01	-0.92	0.29	0.63	-1.64	0.15	0.80	8.15	0.04	0.02	0.49	0.03	0.62
Pyment	-1.36	0.01	-0.10	<0.01	-0.10	0.89	<0.01	-1.18	0.25	0.64	2.51	0.47	0.32	0.16	0.05	0.34
BrainAgeNeXt	-0.66	0.07	0.02	0.26	-0.85	0.23	<0.01	0.99	0.33	<0.01	-5.65	0.10	0.11	0.05	0.08	0.09

Across all models, the age coefficients were consistently negative and significant in 3 out of 4 models, confirming that BAG decreases with age, regardless of dataset, in line with the regression-to-the-mean effect. The magnitude of this effect was large in ENIGMA (largest) and DeepBrainNet, while small for Pyment and particularly almost neglible in BrainAgeNeXt.

Datasets had small effect sizes for all models but for ENIGMA, where the dataset had medium effect sizes. Dataset coefficients reached statistical significance in ENIGMA for both the ADNI and JUK cohorts, and in DeepBrainNet for the JUK cohort. In all cases, the coefficients were positive, indicating that, after adjusting for age, participants from ADNI and JUK tended to exhibit older-appearing brains relative to the reference group (RRIB), according to the ENIGMA and DeepBrainNet estimations respectively. Finally, the regression intercepts were generally negative, indicating a systematic underestimation of brain age when predictions converged toward the center of the age distribution for the RRIB dataset (the dataset with a wider range and more uniform age distribution).

#### 3.3.2 Ethnicity and education related bias

**Fig. 4.**
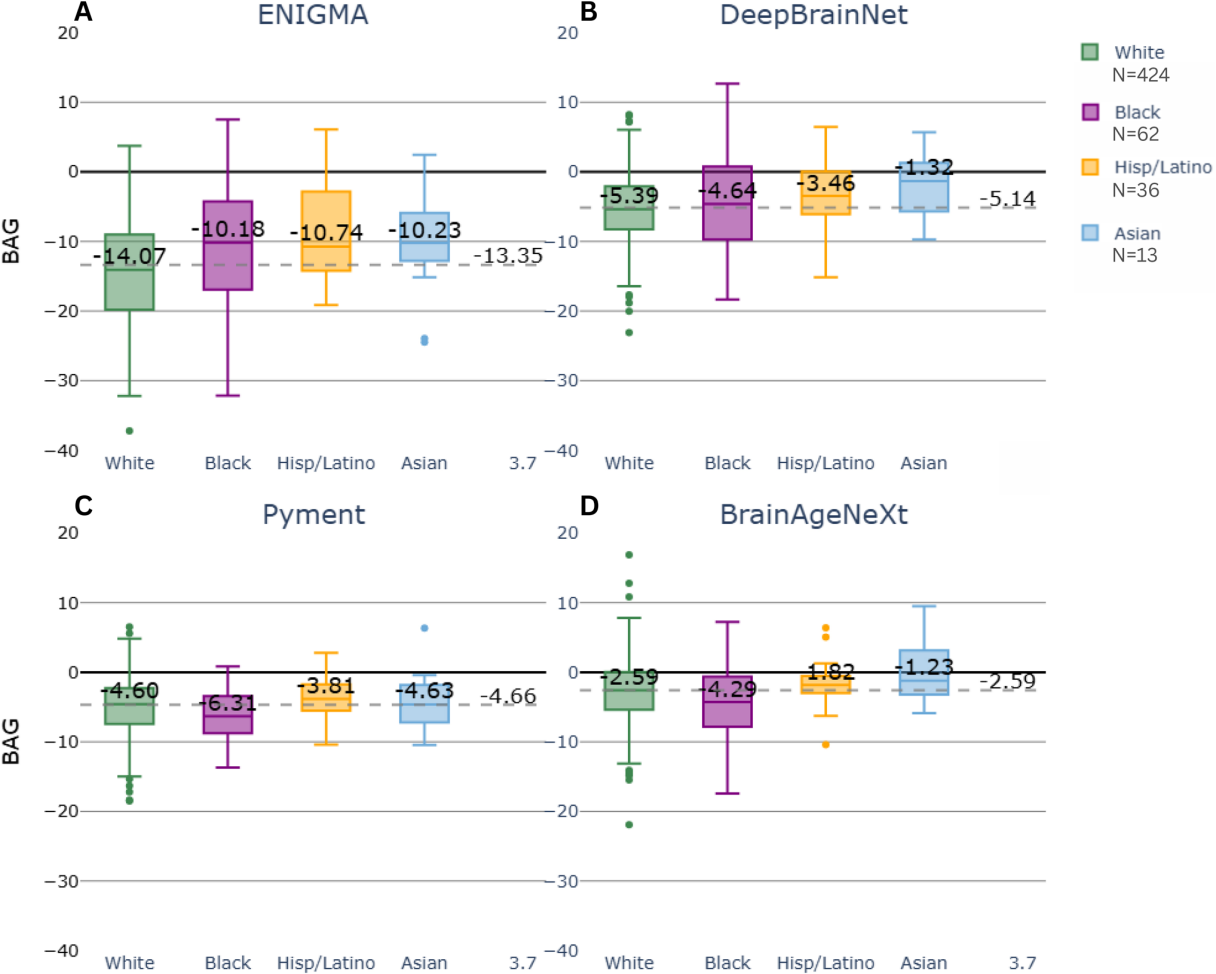
BAG box plots for the four models (A: ENIGMA. B: DeepBrainNet. C: Pyment. D: BrainAgeNeXt) across ethnic groups within cognitively normal (CN) subjects from the ADNI dataset. The dotted line denotes the model-specific median BAG for the entire CN cohort in the dataset.

We assessed model robustness with respect to ethnicity-related bias using the CN participants of the ADNI dataset. Figure 4 shows BAG distributions by ethnicity for each model. While visual inspection suggests relatively consistent medians across groups for Pyment and BrainAgeNeXt, statistical analysis revealed significant effects.

An ANCOVA analysis with ethnicity as categorical variable and age as covariate, revealed that Pyment and BrainAgeNeXt had statistically significant effects of ethnicity, although with small effect sizes. ENIGMA and DeepBrainNet did not show significant ethnicity-related effects. Full results are available in Table 7.

**Table 7.** Statistic F, *p*-values, and effect sizes from the ANCOVA implemented for each model, assessing demeaned age and ethnicity on BAG, evaluated on CN subjects from ADNI cohort.

	Intercept	Age		Ethnicity		Age’s effect sizes	Ethnicity effect sizes
Model	Value	Statistic F	*p*	Statistic F	*p*	Cohen’s f²	Cohen’s f²
ENIGMA	-14.14	101.5	<0.01	0.76	0.52	0.19	0.00
DeepBrainNet	-4.90	200.19	<0.01	1.23	0.30	0.44	0.00
Pyment	-4.81	184.11	<0.01	9.57	<0.01	0.37	0.06
BrainAgeNeXt	-2.51	68.91	<0.01	7.70	<0.01	0.12	0.04

Post-hoc Tukey tests identified specific significant pairwise differences: for Pyment, between Black and Hispanic/Latino participants (*p* = 0.02), and a trend between White and Black (*p* = 0.06); and for BrainAgeNeXt, between White and Black (*p* = 0.02), Black and Hispanic/Latino (*p* = 0.02), and Black and Asian (*p* < 0.01). These findings indicate that while the overall ethnicity effect sizes were small, some pairwise contrasts were statistically significant, particularly for the Black subgroup.

In contrast, no significant associations were found between BAG and the years of education for the ADNI and UNSAM_LC cohorts, which were the only datasets containing this information. Full statistical results are reported in Supplementary Tables S4 and S5 of the Supplementary Material.

### 3.4 Interpretability

**Fig. 5.**
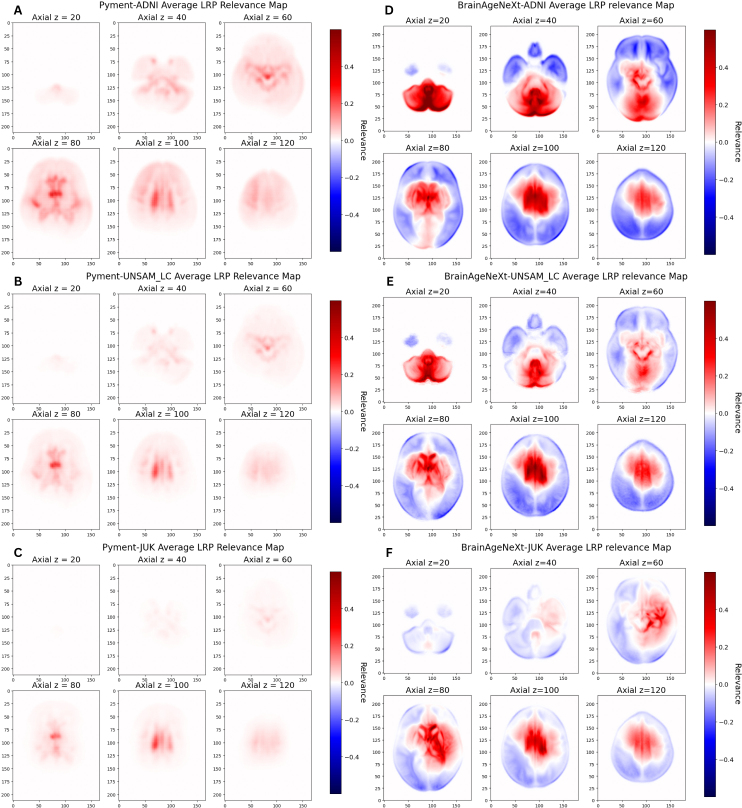
Axial slices of average normalized relevance maps from Z = 20 to Z = 120, obtained with the LRP algorithm for CN individuals evaluated with Pyment (A, B, C for ADNI, UNSAM_LC, and JUK respectively) and BrainAgeNeXt (D, E, F). (For axial, sagittal, and coronal views on LRP average maps from all 4 datasets centered on the most relevant voxel coordinate, see Supplementary Figure S3 of the Supplementary Material, Section S3).

Figure 5 shows average relevance maps generated with the LRP algorithm for CN subjects from the ADNI, UNSAM_LC, and JUK cohort for the 3D CNN based models. In these maps, red voxels indicate regions contributing more strongly and positively to the final age prediction, whereas blue voxels reflect regions with lower or opposing relevance, suggesting minimal or negative contributions to the predicted age.

Visual inspection of the relevance maps in Figure 5 reveals that, across all datasets, the voxels contributing the most strongly to brain age prediction for both 3D CNN-based models are predominantly located in subcortical regions, including the lateral ventricles, thalamus, and basal ganglia. These findings are supported by Table 8, which ranks the average regional relevance scores per dataset using the Hammer’s atlas. The lateral ventricles consistently exhibit the highest relevance values, indicating that these regions are highly attributed by the models when estimating brain age.

**Table 8.** Top 5 ROIs from the Hammer’s atlas with the highest average relevance score across datasets and models based on 3D CNNs.

	ADNI		UNSAM_LC		RRIB		JUK		Total	
Model	ROI	Score	ROI	Score	ROI	Score	ROI	Score	ROI	Score
Pyment	Third-ventricle	0.153	Lateral-ventricle-R^*^	0.133	Lateral-ventricle-R^*^	0.132	Lateral-ventricle-R^*^	0.116	Lateral-ventricle-R^*^	0.533
	Lateral-ventricle-R^*^	0.151	Third-ventricle	0.097	Caudate-nucleus-R	0.103	Lateral-ventricle-L^*^	0.074	Third-ventricle	0.402
	Substantia-nigra-R	0.137	Lateral-ventricle-L^*^	0.093	Lateral-ventricle-L^*^	0.101	Thalamus-R	0.068	Lateral-ventricle-L^*^	0.392
	Thalamus-R	0.126	Thalamus-R	0.090	Third-ventricle	0.091	Third-ventricle	0.060	Caudate-nucleus-R	0.369
	Lateral-ventricle-L^*^	0.123	Caudate-nucleus-R	0.089	Thalamus-R	0.085	Corpus-callosum	0.059	Thalamus-R	0.369
BrainAgNeXt	Cerebellum-R	0.299	Lateral-ventricle-R^*^	0.473	Lateral-ventricle-R^*^	0.475	Lateral-ventricle-R^*^	0.425	Lateral-ventricle-R^*^	1.605
	Cerebellum_L	0.260	Lateral-ventricle-L^*^	0.384	Lateral-ventricle-L^*^	0.353	Pallidum-R	0.286	Lateral-ventricle-L^*^	1.178
	Third-ventricle	0.255	Corpus-callosum	0.332	Third-ventricle	0.319	Insula-posterior-long-gyrus-R	0.284	Third-ventricle	1.09
	Caudate-nucleus-L	0.243	Third-ventricle	0.311	Corpus-callosum	0.302	Thalamus-R	0.274	Corpus-callosum	1.079
	Lateral-ventricle-R^*^	0.231	CG-posterior-Cingulate-gyrus-R	0.300	Thalamus-R	0.288	Insula-anterior-long-gyrus-R	0.267	Thalamus-R	0.994

* Lateral-Ventricle R and L labels correspond to the Lateral-ventricle-excluding-temporal-horn-R/ L ROIs respectively.

Notably, in the relevance maps generated by BrainAgeNeXt (Fig. 5D–F), a trend emerges whereby datasets with older age distributions (e.g., ADNI in D) show increased relevance in inferior brain regions, particularly the cerebellum. This may reflect model adaptation to age-related structural changes in older cohorts.

**Fig. 6.**
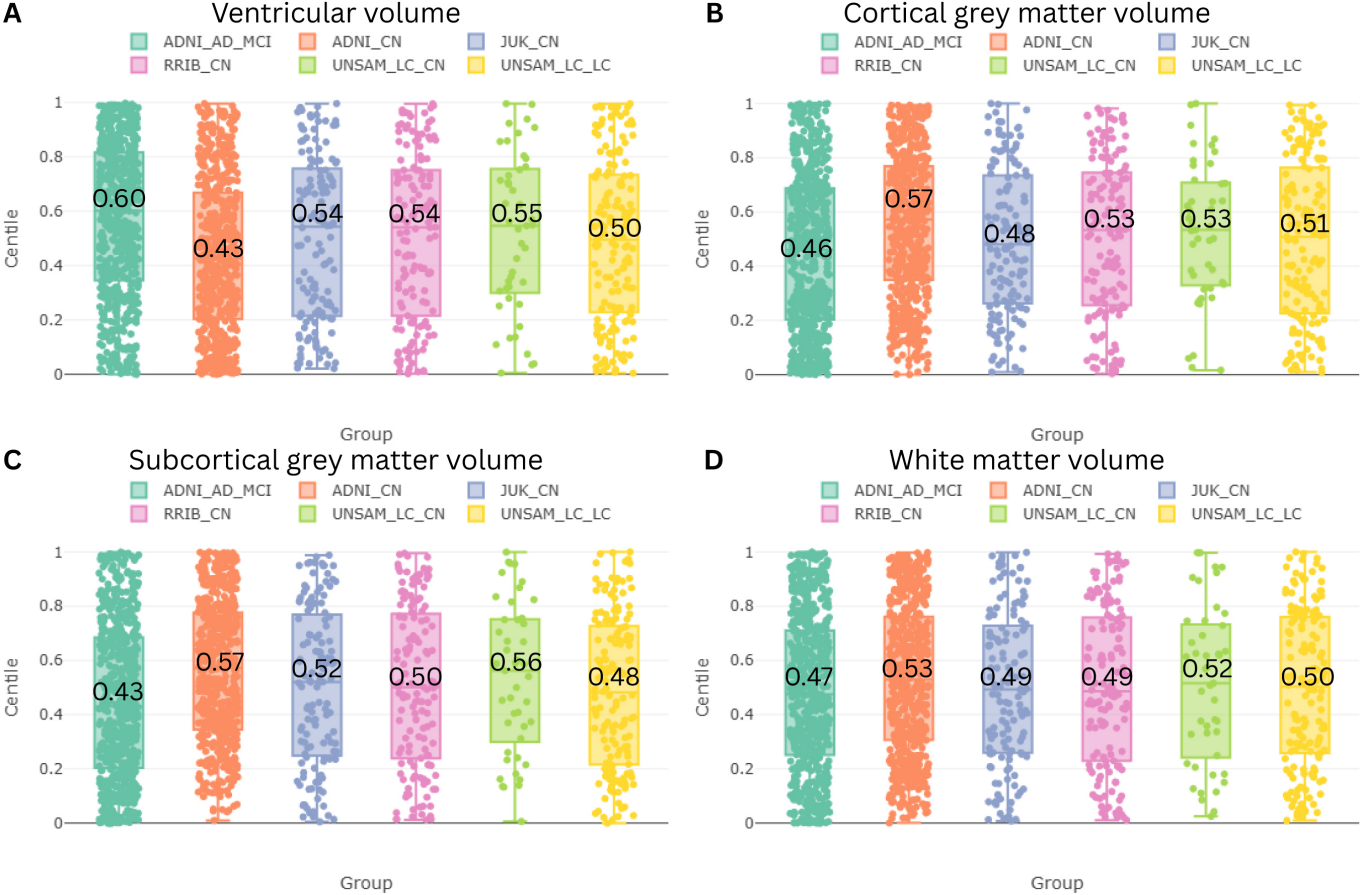
Individualized centile scores by dataset and diagnostic group relative to normative expectations for Ventricular (A), Grey matter (B), Subcortical grey matter, (C) and White matter volume (D) obtained from the Brainchart Web Application.

To further investigate anatomical correlates of BAG, we assessed centile-normalized tissue volumes using the BrainChart platform (Fig. 6). Centile scores were derived for ventricular volume, total cortical grey matter, subcortical grey matter, and white matter across all datasets, including clinical subgroups, enabling comparison with normative age-matched trajectories.

As expected, individuals with MCI or AD in the ADNI cohort exhibited larger ventricular volumes and reduced grey matter volumes relative to normative centiles, consistent with patterns of accelerated brain aging. Interestingly, CN individuals from ADNI showed smaller-than-average ventricular volumes and preserved grey matter, suggesting that this subgroup may represent a particularly healthy aging cohort.

In the remaining datasets, ventricular volumes showed slight median shifts above the 50th centile, while grey matter volumes generally aligned with normative expectations. No consistent deviations were observed in white matter across cohorts.

### 3.5 Group-level differences and statistics

**Fig. 7.**
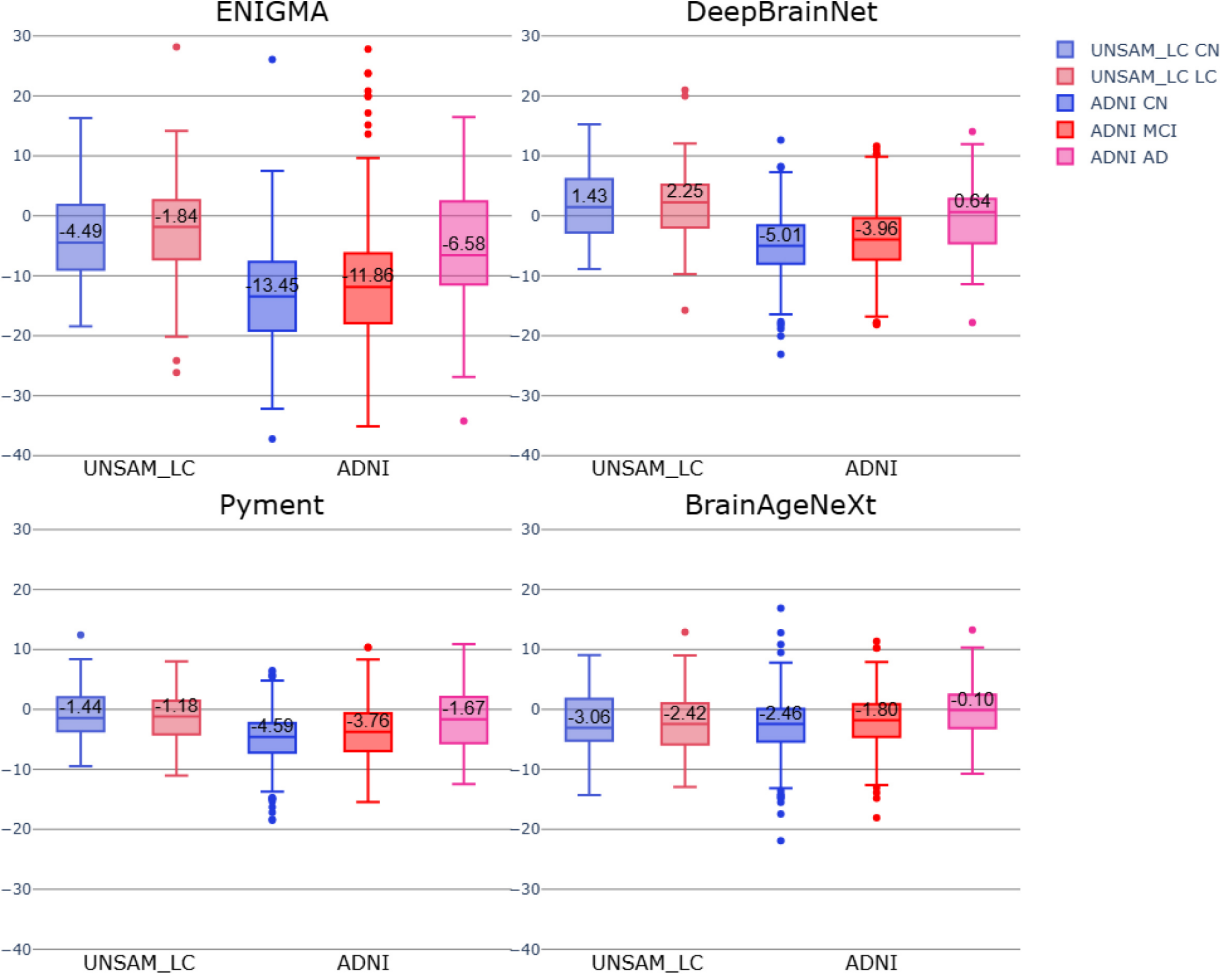
BAG box plots for the four models (A: ENIGMA. B: DeepBrainNet. C: Pyment. D: BrainAgeNeXt) evaluated on clinical subgroups and CN participants from the ADNI and UNSAM_LC cohorts. The group and model-specific medians BAG are shown for each box.

We assessed whether clinical subgroups exhibited significantly higher brain age gap (BAG) values compared to cognitively normal (CN) individuals, as would be expected in cases of accelerated brain aging. Figure 7 shows boxplots of BAG for each model across the ADNI and UNSAM_LC datasets and their respective subgroups.

An ANCOVA was performed to assess the effect of clinical groups on BAG, adjusting for age and sex. Significant group effects were observed in the ADNI cohort for all models (see Supplementary Table S6). Post-hoc Tukey tests revealed significant pairwise differences among all three ADNI subgroups (CN, MCI, AD), as reported in Table 9.

**Table 9.** *p*-Values from post-hoc Tukey tests following significant ANCOVA results on clinical subgroups from the ADNI cohort (CN vs. MCI vs. AD).

	ADNI (CN vs MCI)	ADNI (CN vs AD)	ADNI (MCI vs AD)
Model	*p*-Value	*p*-Value	*p*-Value
ENIGMA	<0.001	<0.001	<0.001
DeepBrainNet	0.033	<0.001	<0.001
Pyment	<0.001	<0.001	0.002
BrainAgeNeXt	0.004	<0.001	0.002

In contrast, for the UNSAM_LC cohort, individuals in the Long COVID subgroup exhibited slightly higher BAG values on average, but no statistically significant differences were found between the LC and CN groups (see Supplementary Table S7).

## 4 Discussion

In this study, we systematically evaluated the accuracy, bias, and biomarker utility of four publicly available brain age prediction models across four external MRI datasets representing diverse populations and acquisition protocols. Our findings confirm that models based on CNNs consistently outperformed traditional ML approaches in terms of accuracy. However, all models exhibited age-dependent biases, particularly a tendency to underestimate brain age in older individuals, which may limit their utility as standalone biomarkers in aging populations, a key target group for clinical applications. Despite these limitations, comparisons between clinical and CN groups revealed significant BAG differences in several models, suggesting that brain age prediction can still serve as a useful biomarker of pathological aging when matched healthy control groups are available for comparison.

### 4.1 Accuracy evaluation

In this benchmark of publicly available pre-trained models, the two 3D CNN-based approaches showed the best performance in terms of prediction accuracy and consistency across datasets. When comparing the two models, BrainAgeNeXt achieved the lowest overall MAE, despite Pyment being trained on a substantially larger and more heterogeneous dataset spanning multiple acquisition sites and age ranges. This result may reflect differences in modeling strategies: BrainAgeNeXt is built on ConvNeXt blocks, a recent deep learning architecture inspired by transformer models, and applies a post-hoc bias correction strategy that improves age-related accuracy metrics.

However, BrainAgeNeXt’s original reported accuracy was only replicated in the JUK dataset, which closely matches its training distribution in terms of age (predominantly adolescents and young adults). In contrast, Pyment exhibited a more stable performance across all datasets, closely matching the accuracy reported in its original publication, and suggesting stronger generalization capabilities, likely driven by its broader and larger training set.

Taken together, these findings suggest that differences in training set size, age coverage, and modeling choices (including post-hoc correction) all influence model performance in real-world scenarios. While novel architectures may improve performance under specific conditions, training data diversity remains a critical factor for achieving generalizability in brain age prediction.

### 4.2 Bias and robustness

Consistent with prior reports (Cole & Franke, 2017), all evaluated models exhibited a regression-to-the-mean effect, whereby predicted brain ages tend to gravitate toward the center of the training age distribution. Specifically, predictions were consistently underestimated in the ADNI dataset and overestimated in the JUK dataset, reflecting the demographic composition of these cohorts: ADNI predominantly comprises older adults (approximately 70 years of age), whereas JUK primarily includes young adults and adolescents. Among all models, BrainAgeNeXt showed the greatest mitigation of this effect, likely due to its use of a post-hoc linear correction strategy (Beheshti et al., 2019).

After incorporating age-dataset interaction terms in our regression analysis, we found that dataset membership was no longer a significant predictor of BAG, suggesting that the previously observed dataset-specific biases (Table 6) were largely driven by age differences across cohorts. This indicates that, despite differences in geographical origin, the models did not systematically assign older or younger brain ages solely based on dataset origin.

The low dataset-related bias observed is notable given that preprocessing pipelines across models only included basic standardization steps such as bias field correction and spatial normalization to the MNI152 template. No specific harmonization strategies, such as ComBat, were applied. However, it is important to note that structural T1-weighted MRI protocols are generally standardized across large neuroimaging databases, potentially reducing the necessity for harmonization in this context, especially when compared to other neuroimaging modalities.

Nevertheless, the more accurate models (Pyment and BrainAgeNeXt) exhibited statistically significant differences in BAG across ethnic groups, although with small effect sizes, highlighting their sensitivity to ethnicity-related biases. In contrast, for ENIGMA and DeepBrainNet, such effects were likely masked by their lower prediction accuracy. This analysis was only possible in the ADNI dataset, which includes older adults and was the only cohort with ethnicity information available. These effects likely reflect the demographic imbalances in large-scale neuroimaging datasets, where individuals from White ethnic backgrounds are disproportionately represented. Overall, age-related bias, rather than dataset or ethnicity per se, remains the main limitation for cross-population robustness.

These results underscore the importance of bias correction techniques to enhance the clinical interpretability of brain age predictions. For instance, BrainAgeNeXt applies a linear regression-based adjustment to predicted values (Beheshti et al., 2019), which helps to reduce, but not fully eliminate, the regression-to-the-mean effect. Notably, this correction and its generally young training set introduces a small but systematic underestimation bias across the age spectrum.

Other mitigation strategies include training-time interventions such as reweighting the loss function by age group or or stratified model training across age bins, allowing the model to give greater importance to underrepresented age ranges, thereby reducing systematic prediction errors across the lifespan. Furthermore, residualization approaches can be applied post hoc to remove age dependence from the BAG, though these require careful application to avoid removing signals of interest in clinical populations. We emphasize that any correction method should be transparently reported and validated to ensure consistent performance across age groups.

### 4.3 Interpretability

Beyond predictive performance, we investigated model interpretability by generating average relevance maps using the LRP algorithm for the models based on 3D CNNs. These analyses revealed that voxels located in subcortical regions of the brain were consistently assigned high relevance in the brain age prediction models. In particular, the lateral ventricles exhibited the highest average relevance scores across both models, as quantified using ROI-level analysis based on the Hammer’s atlas. These findings align with previous studies highlighting the importance of central brain regions, particularly ventricular enlargement, as key anatomical drivers in brain age prediction models (Hepp et al., 2021; Hofmann et al., 2022).

It is important to note that the LRP relevance maps for Pyment were obtained using the original model-specific implementation provided by the authors, while for BrainAgeNeXt, we employed a generalized approach based on the Zennit library. These methodological differences may partly explain the variation in relevance map intensities observed across models.

In the case of the BrainAgeNeXt model, we also found that datasets with older age distributions showed increased attribution in inferior brain regions, particularly the cerebellum. This pattern suggests that as the brain ages, voxels in these lower regions become progressively more relevant, at least for this model. These results align with evidence indicating that cerebellar volume begins to decline around the mid-fifties in humans (Luft, 1999).

To further investigate age-specific biases, we used the BrainChart platform to derive tissue-specific centile scores for ventricular, cortical gray matter, subcortical gray matter, and white matter volumes. As expected, clinical subgroups from ADNI exhibited ventricular volume centiles well above the median, consistent with accelerated aging profiles. In contrast, CN individuals from ADNI displayed ventricular volume centiles below the 50th percentile, suggesting a younger than average brain phenotype relative to population norms.

Considering that the lateral ventricles were identified as the most relevant anatomical region for brain age prediction across models, this observation suggests that the systematic underestimation of brain age in ADNI CN subjects may not simply reflect a regression-to-the-mean artifact. Instead, it likely captures a genuine anatomical phenotype of decelerated brain aging, potentially driven by strict inclusion criteria in ADNI, which select for individuals with exceptionally healthy aging profiles. These findings emphasize the importance of considering recruitment criteria and sample representativeness when using CN cohorts to train brain age models, as such cohorts may not reflect the true population distribution of healthy aging; implying that there is a risk of overfitting brain age models to “super-normal” control populations, such as those found in highly selective research cohorts like ADNI. If used for model training, such cohorts may bias predictions and reduce generalizability to broader, more representative populations.

### 4.4 Group-level differences and statistics

At the group-level, we evaluated BAG differences between clinical and control subgroups in the UNSAM_LC and ADNI datasets. As expected, AD patients exhibited substantially higher BAG values compared to CN individuals across all models, consistent with prior studies showing accelerated brain aging in Alzheimer’s disease (Cole & Franke, 2017; Singh et al., 2022). MCI patients also showed significantly elevated BAG values relative to CN individuals, suggesting early signs of accelerated aging. Moreover, the four models detected a significant difference between MCI and AD patients, highlighting the importance of model sensitivity and accuracy when distinguishing between groups with more subtle phenotypic differences.

In contrast, within the UNSAM_LC dataset, no significant BAG differences were detected between LC patients and their matched CN counterparts. While median BAG was slightly elevated in the LC group, this difference was not statistically significant. These findings are consistent with prior results from this cohort, which showed normal cognitive performance and only mild regional atrophy in LC individuals. While BAG does not currently differentiate these groups, it may still provide value for longitudinal monitoring, especially as subtle structural or functional changes may emerge over time.

Taken together, these results suggest that current brain age prediction models are not yet suitable for individual-level assessment of pathological brain aging. This limitation arises because systematic age-related biases lead to negative BAG values in older individuals, including those with clear neurodegenerative pathology, where positive BAG values would be expected. As a consequence, a single individual’s BAG cannot be reliably interpreted in isolation. In contrast, when applied to matched groups, these biases are shared across groups and therefore largely cancel out, allowing BAG to function as a meaningful group-level biomarker that captures population-level deviations from normative aging trajectories.

### 4.5 Limitations

This study has several limitations that should be considered when interpreting the results. A key limitation of this study is that the evaluated models differ not only in architecture but also in training data scale, population heterogeneity, and the use of post-hoc bias correction techniques. As a result, we cannot disentangle the individual contributions of these factors to overall performance. For example, the better generalization of Pyment (i.e., its measured accuracy was closely matched what was originally reported) may reflect its use of a substantially larger and more diverse training set, compared to BrainAgeNext (its measured accuracy was higher than originally reported). On the other hand, BrainAgeNeXt applies a linear post-hoc correction that improves bias metrics. Nonetheless, we aimed to reduce confounding factors in our evaluation by testing the models on diverse independent datasets covering a wide adult age range (18–90 years), from multiple countries and protocols. Furthermore, we computed balanced MAE and ME metrics using matched sample sizes to allow a fairer comparison of model generalizability. These steps help mitigate, although not eliminate, the impact of training differences, and underscore the need for future standardized benchmarking frameworks.

Second, although we assessed and modeled biases across datasets and age groups, many of the datasets exhibited narrow or skewed age distributions (e.g., JUK with young adults; ADNI with older adults). This complicates efforts to fully disentangle age-related biases from dataset-specific effects, even when using age-dataset interaction terms in our regression models. A more balanced age representation across datasets would improve the ability to isolate and correct model biases.

Third, it is important to note that some datasets, such as ADNI, apply strict cognitive and health-related inclusion criteria for their control groups, potentially selecting individuals with unusually healthy aging profiles. This may limit generalizability and should be considered when interpreting BAG values across cohorts.

Fourth, the analysis of ethnicity-related bias was only possible in the ADNI dataset, which predominantly includes older adults. While this limits broader generalization, our findings do suggest that ethnicity remains a relevant source of variability in model performance and should be explicitly modeled in future studies.

Finally, while our analyses focused on publicly available brain age models, we did not account for site- or scanner-specific factors such as field strength, voxel resolution, or manufacturer differences, which have been shown to influence model performance in multi-site settings. Although most datasets used 3T MRI and comparable protocols, future work should explicitly model or harmonize scanner effects to further reduce technical bias and improve generalizability.

## 5 Conclusion

This study provides a comprehensive evaluation of current brain age prediction models across diverse populations, highlighting both their strengths and limitations. In our benchmark, models based on 3D CNNs (Pyment and BrainAgeNeXt) demonstrated greater accuracy, reduced variance, and less pronounced age-related bias, particularly when trained on large, heterogeneous datasets. Interpretability analyses revealed consistent attribution patterns across models, with subcortical structures, especially the lateral ventricles, being consistently assigned high relevance in age prediction. Notably, in older cohorts, the more advanced model with transformer-inspired mechanisms (BrainAgeNeXt) also identified the cerebellum as a relevant structure, suggesting age-specific differences in the anatomical basis of predictions. Additionally, our analysis of ethnicity within the ADNI cohort revealed statistically significant but small differences in BAG for some models, underscoring their sensitivity to demographic biases. This reinforces the importance of improving the representativeness of training datasets, particularly with respect to underrepresented ethnic groups. By comparing anatomical tissue volumes to large normative databases, we demonstrated the value of contextualizing brain age metrics within population-level neuroanatomical variation to detect biases. Taken together, our findings support the use of the brain age gap as a promising group-level biomarker of brain aging, while underscoring the need for bias correction and careful cohort selection before it can be reliably applied at the individual level in clinical practice.

## Supplementary Material

Supplementary Material

## Data Availability

All code used for image preprocessing, model evaluation, and statistical analysis, as well as the resulting brain age predictions for all models and datasets, are publicly available at: https://github.com/mabelzunce/brainage-models-benchmark
