# Supplementary material for: Bias and generalizability of brain age prediction models: A multi-cohort evaluation with anatomical and interpretability insights
Source: Imaging Neurosci (Camb). 2026 Mar 12;4:IMAG.a.1164. doi: 10.1162/IMAG.a.1164 (PMC12983579; doi:10.1162/IMAG.a.1164)
Supplement: Supplementary Material [file IMAG.a.1164_supp.pdf]

Supplementary Material for “Bias and Generalizability of Brain Age Prediction Models: A Multi-Cohort Evaluation with Anatomical and Interpretability Insights”

Lautaro J. Aguzin Parrilli, Martin A. Belzunce

S1. Image Quality Assessment

Figure S1 shows the distribution of two image quality metrics, Contrast-to-Noise Ratio (CNR) and Entropy Focus Criterion (EFC), for each dataset. Table S1 presents the Spearman correlation between CNR and RFC, and the absolute error in age predictions for each model, to assess if image quality impacts the model's accuracy. No significant correlation was found between absolute prediction error and image quality metrics.

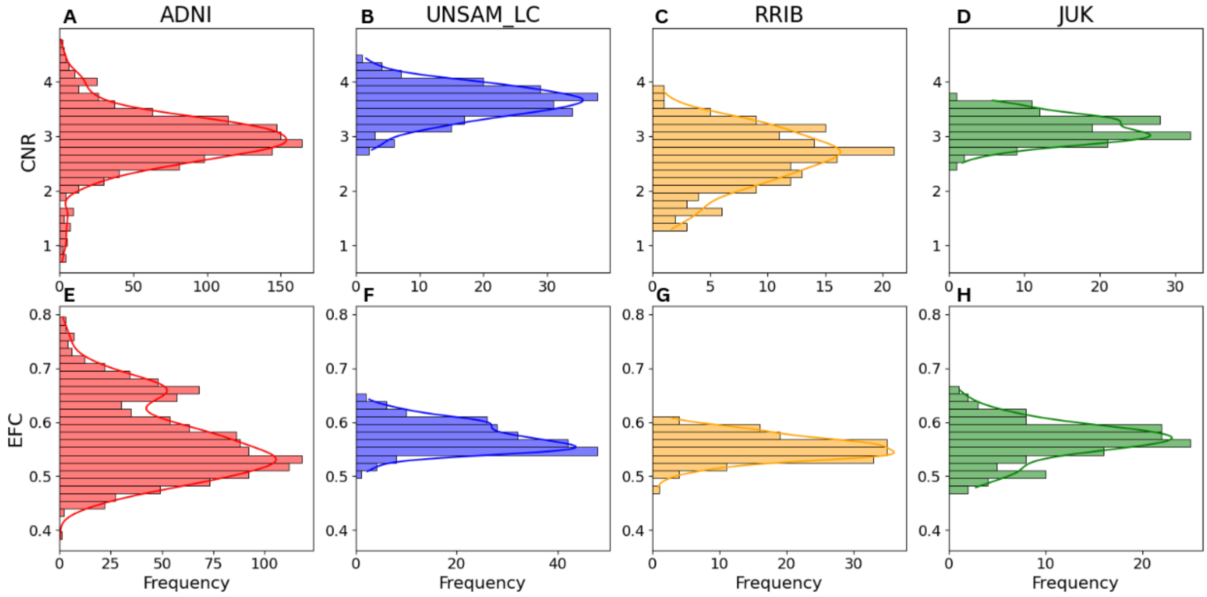

Figure S1. *Contrast-to-Noise Ratio (CNR) and Entropy Focus Criterion (EFC) distributions across datasets: ADNI (A,E), UNSAM_LC (B,F), RRIB (C,G), and JUK (D,H) respectively.*

Table S1. *Spearman’s correlation coefficients between CNR and EFC image quality metrics and model predictions’ absolute error for each dataset, with their p-Values and effect sizes.*

Model	CNR			EFC		
	coef	p-Value	Effect size	coef	p-Value	Effect size
**ENIGMA**	-0.102	0.002	0.010	-0.062	0.064	0.003
**DeepBrainNet**	-0.023	0.510	<0.001	0.006	0.858	<0.001
**Pyment**	-0.090	0.008	0.008	<0.001	0.996	<0.001
**BrainAgeNeXt**	-0.016	0.630	<0.001	-0.001	0.969	<0.001

S2. Comparison between DeepBrainNet preprocessing methods

We compared the mean absolute error (Table S3) and mean error (Table S4) of the DeepBrainNet model for three different implementations. The implementation of their public repository (https://github.com/vishnubashyam/DeepBrainNet/tree/master) features brain extraction performed with synthstrip (Figure S2-A) and Bet (Figure S2-C), as well as preprocessing included in the ANTsPyNet package. The original DeepBrainNet repository with BET in the preprocessing achieved the best accuracy.

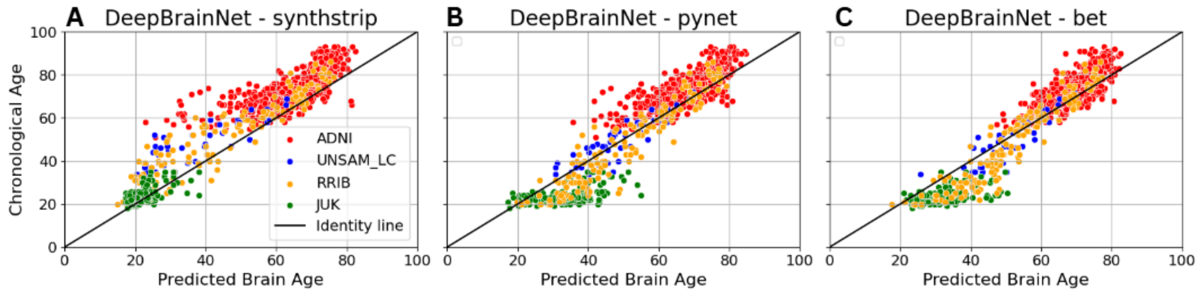

Figure S2. *Scatter plots of predicted vs. chronological brain age for CN subjects across all datasets, using different skull-stripping methods in the DeepBrainNet pipeline. A) synthstrip. B) Through the ANTsPyNet utility (pynet). C) BET.*

Table S2. *Mean Absolute Error for the DeepBrainNet model tested with different skull-stripping tools across external datasets (only CN subjects) and balanced global ME (mean ± SD) computed from 20 random subsamples of 47 CN participants per dataset.*

	ADNI	UNSAM_LC	RRIB	JUK	Balanced global MAE*
**DeepBrainNet-Synthstrip**	9.16	11.24	7.15	3.24	7.67
**DeepBrainNet-pynet**	6.60	4.19	5.34	9.93	6.49
**DeepBrainNet-BET**	5.87	4.67	6.00	8.39	6.34

*Balanced global MAE was computed by averaging 20 random subsamples of 47 cognitively normal participants per dataset, drawn without replacement

Table S3. *Mean Error for the DeepBrainNet model tested with different skull-stripping tools across external datasets (only CN subjects) and balanced global ME (mean ± SD) computed from 20 random subsamples of 47 CN participants per dataset.*

	ADNI	UNSAM_LC	RRIB	JUK	Balanced global ME*
**DeepBrainNet-Synthstrip**	-8.82	-11.12	-6.52	-2.04	-7.1
**DeepBrainNet-pynet**	-5.76	-0.73	2.21	9.51	1.25
**DeepBrainNet-BET**	-4.79	1.51	2.79	8.26	2.07

*Balanced global ME was computed by averaging 20 random subsamples of 47 cognitively normal participants per dataset, drawn without replacement

S3. Explainability Maps

Explainability maps of sagittal, coronal and axial views centred on the voxel of maximum relevance.

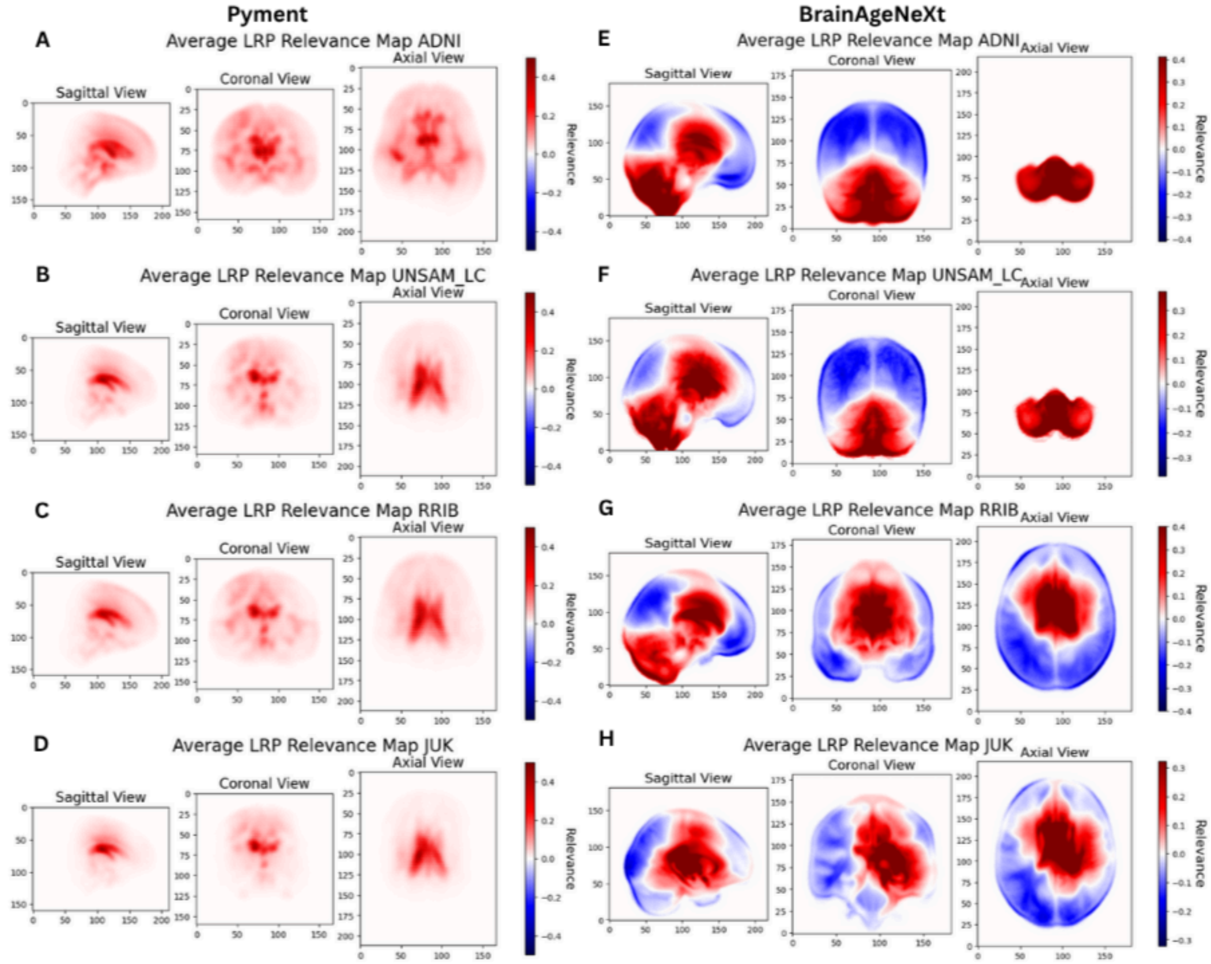

Figure S3. *Sagittal, coronal and axial slices of average relevance maps centred at the most relevant voxel coordinate, obtained with the LRP algorithm for CN individuals from each dataset for Pyment (A,B,C,D for ADNI, UNSAM_LC, RRIB and JUK, respectively) and BrainAgeNeXt (E, F, G, H).*

S4. Effect of Educational Level on BAG Predictions

Tables S5 and S6 show results for fitted multivariate linear regression models for BAG as the dependent variable and age, years of education level and clinical groups as predictors, for ADNI and UNSAM_LC datasets, respectively. Education level had a negligible effect on all models.

Table S4. *Coefficients, p-values, effect sizes, and R^2 from multivariate regressions with BAG as the dependent variable and demeaned age, years of education, and clinical group (using the CN group as reference) as predictors, evaluated on the full ADNI sample for each model.*

Model	Intercept		Age		Years of education		Group (MCI)		Group (AD)		Age's effect size	Education effect size	R^2
	β_0	*p*	β_1	*p*	β_2	*p*	β_3	*p*	β_4	*p*	Cohen's f^2	Cohen's f^2	
**ENIGMA**	-13.70	<0.01	-0.50	<0.01	-0.02	0.87	2.28	<0.01	8.52	<0.01	0.25	0.00	0.24
**DeepBrainNet**	-4.93	<0.01	-0.39	<0.01	<0.01	0.89	1.34	<0.01	5.39	<0.01	0.49	0.00	0.36
**Pyment**	-5.96	<0.01	-0.30	<0.01	0.04	0.35	1.46	<0.01	4.10	<0.01	0.39	0.00	0.31
**BrainAgeNeXt**	-3.25	<0.01	-0.21	<0.01	0.02	0.62	1.13	<0.01	3.16	<0.01	0.17	0.00	0.17

Table S5. *Coefficients, p-values, effect sizes, and R^2 from multivariate regressions with BAG as the dependent variable and demeaned age, years of education, and clinical group membership (using the CN group as reference) as predictors, evaluated on the full UNSAM_LC cohort for each model.*

Model	Intercept		Age		Years of education		Group (COVID)		Age's effect size	Education effect size	R^2
	β_0	*p*	β_1	*p*	β_2	*p*	β_3	*p*	Cohen's f^2	Cohen's f^2	
**ENIGMA**	0.90	0.71	-0.42	<0.01	-0.31	0.06	1.16	0.34	0.29	0.00	0.23
**DeepBrainNet**	-8.03	<0.01	0.12	0.05	-0.24	0.18	-0.36	0.78	0.02	0.01	0.03
**Pyment**	-0.96	0.51	-0.01	0.71	<0.01	0.99	-0.48	0.51	0.00	0.00	<0.01
**BrainAgeNeXt**	-0.17	0.92	0.20	<0.01	-0.15	0.19	0.04	0.96	0.13	0.00	0.13

S5. Group Level Analysis

Table S6. *F-statistics, p-values, and effect sizes from ANCOVA models assessing BAG differences between clinical groups (Long COVID vs. CN) in the UNSAM cohort, for each brain age prediction model. The models included demeaned age, clinical group, sex, and years of education as covariates. Effect sizes are reported for demeaned age and clinical group.*

Model	Intercept	Age		Group		Sex		Years of education		Age's effect size	Group's effect size
	Value	Statistic F	*p*	Statistic F	*p*	Statistic F	*p*	Statistic F	*p*	Cohen's f^2	Cohen's f^2
**ENIGMA**	-4.98	49.77	<0.01	0.95	0.33	0.03	0.85	0.66	0.72	0.27	0.00
**DeepBrainNet**	1.29	49.51	<0.01	0.30	0.58	0.04	0.83	1.21	0.29	0.26	0.00
**Pyment**	1.43	0.72	0.39	0.19	0.66	1.80	0.18	1.49	0.16	0.00	0.00
**BrainAgeNeXt**	-1.81	20.61	<0.01	0.00	0.94	2.40	0.12	1.50	0.16	0.11	0.00

Table S7. *F-statistics, p-values, and effect sizes from ANCOVA models assessing BAG differences between clinical groups (AD, MCI, CN) in the ADNI cohort, for each brain age prediction model. The models included demeaned age, clinical group, sex, and years of education as covariates. Effect sizes are reported for demeaned age and clinical group.*

Model	Intercept	Age		Group		Sex		Ethnicity		Years of education		Age's effect size	Group's effect size
	Value	Statistic F	*p*	Statistic F	*p*	Statistic F	*p*	Statistic F	*p*	Statistic F	*p*	Cohen's f^2	Cohen's f^2
**ENIGMA**	-4.83	277.24	<0.01	35.08	<0.01	1.68	0.19	1.08	0.37	0.04	0.83	0.25	0.06
**DeepBrainNet**	-1.26	474.5	<0.01	28.02	<0.01	9.18	0.03	2.37	0.04	0.13	0.71	0.52	0.06
**Pyment**	1.17	431.70	<0.01	33.70	<0.01	0.05	0.81	5.84	<0.01	0.38	0.54	0.41	0.06
**BrainAgeNeXt**	1.81	194.69	<0.01	20.60	<0.01	3.40	0.06	5.04	<0.01	0.22	0.64	0.17	0.03
